# Risk perception of sun exposure and knowledge of vitamin D among the healthcare providers in a high-risk country: a cross-sectional study

**DOI:** 10.1186/s12909-023-04001-0

**Published:** 2023-01-20

**Authors:** Mahbubul H. Siddiqee, Badhan Bhattacharjee, Mahbub Hasan, Mohammad Shojon, Mehedi Hassan, Rashawan Raziur Rouf, Umme Raihan Siddiqi, Fazlay Rabbi, Umme Ruman Siddiqi

**Affiliations:** 1 Department of Mathematics and Natural Sciences, Microbiology Program, School of Data and Sciences, BRAC University, 1212 Dhaka, Bangladesh; 2 Research Wing, Red & White Innovations. Mirpur DOHS, 1216 Dhaka, Bangladesh; 3 Biomedical Research Foundation, 1230 Dhaka, Bangladesh; 4 Dhaka Medical College and Hospital, 1000 Dhaka, Bangladesh; 5 Department of Biochemistry and Food Analysis, Patuakhali Science and Technology University, 8602 Patuakhali, Bangladesh; 6 Shaheed Suhrawardy Medical College and Hospital, 1207 Dhaka, Bangladesh; 7 Communicable Disease Control (CDC) Unit, Directorate General of Health Services, 1212 Dhaka, Bangladesh

**Keywords:** Vitamin D, Sunlight, Knowledge, Attitude, Practice, KAP, Physicians, Medical students

## Abstract

**Background:**

High levels of vitamin D deficiency are commonly reported even in regions with abundant sunshine. This necessitates a comprehensive understanding of the determinants that influence sun exposure practices. As the primary source of health-related knowledge for the general public, the attitude of the healthcare professionals towards sunlight and their awareness related to vitamin D deficiency can be critical in this regard.

**Methods:**

A cross-sectional survey was conducted among 2,242 physicians, intern doctors, and senior medical students in Bangladesh from October 2019 to February 2020. A pre-tested structured questionnaire (containing twelve close-ended questions) was used. The perceptions of health risks due to sun exposure, and basic knowledge of the physiological and epidemiological aspects of vitamin D deficiency were tested.

**Results:**

An overall negative attitude towards sunlight in the context of Bangladesh was highlighted – 68% participants thought regular sun exposure would be harmful or very harmful; 26% thought the level of UV radiation was very high; 44% recommended using sunscreen always; skin burns, heat stroke, and cancer were selected as potential consequences of regular sun exposure by 45%, 21%, and 30% respondents respectively. Overall knowledge regarding vitamin D deficiency appeared to be biased towards bone health; other symptoms and associated illnesses not having obvious link to Calcium-metabolism were identified much lesser frequently. Furthermore, ‘sunrise to 10 am’ was identified as the best time to get vitamin D by 69% participants; 60% believed < 30 min of weekly sun exposure would be sufficient for the Bangladeshi population; an only 33% identified that prevalence of vitamin D insufficiency in Bangladesh would be 50% or more. Taking vitamin D-rich food was suggested by more respondents over regular sun exposure (43% vs. 33%) as more effective remedial strategy to curb vitamin D deficiency in Bangladesh.

**Conclusion:**

In addition to highlighting some crucial knowledge gaps, results from this study provides a comprehensive baseline dataset for knowledge and attitude regarding the public health aspects of vitamin D deficiency among the healthcare providers in Bangladesh, which would be generalizable to other countries with similar socio-demographic context, and will facilitate taking more effective policies worldwide.

**Supplementary Information:**

The online version contains supplementary material available at 10.1186/s12909-023-04001-0.

## Background

Maintaining an adequate level of vitamin D is essential for the normal functioning of the human body [1]. While this vitamin can also be obtained from dietary sources, it is mostly synthesized naturally in the human body under sunlight, hence frequently called the 'sunshine vitamin' [2]. When human skin is exposed to ultraviolet B (UVB) fraction of the sunlight (290–320 nm wavelength), converted 7-dehydrocholesterol (a precursor molecule for vitamin D synthesis) is into vitamin D following a series of reactions [1, 3].

Due to its wide-ranging effects, deficiency of vitamin D can have a detrimental impact on human health. Various observational and epidemiological studies have reported that vitamin D deficiency may act as a predisposing factor for the development of several kinds of infectious, non-infectious, and bone-related disorders in the human body (e.g., diabetes, cardiovascular disease, colon and breast cancer, Parkinson’s disease, multiple sclerosis, tuberculosis, respiratory infection, osteoporosis, rickets, rheumatoid arthritis, etc.) [1, 2, 4].

An estimated 1 billion people are affected by vitamin-D deficiency and 50% of the world population is affected by Vitamin-D insufficiency around the globe [1, 2]. Despite sunlight being known to be the most important source of vitamin D, a high deficiency of this vitamin is frequently reported in countries where sunshine is abundant [5–7]. Therefore, beyond the availability of sunlight, there must be other factors that influence the population-level vitamin D.

In this regard, while factors like skin complexion and practices like the extent of clothing, usage of sun-protection items, and timing plus duration of direct sunlight exposure are among the directly influencing factors, attitude towards sunlight can strongly influence the practices [8, 9]. Additionally, all these practices and attitudes towards sunlight can be directly or indirectly influenced by the knowledge regarding the harmful and/or beneficial roles of sunlight exposure [10, 11]. Furthermore, the extent and accuracy of knowledge regarding the critical physiological roles of vitamin D, the role of sunlight in its metabolism, the minimum level of sunlight exposure needed for the production of vitamin D in the human body, the best time in the day for maximum production of this vitamin, the symptoms of deficiency, etc. could be crucial determinants of sunlight practices and hence, of vitamin D levels at an individual level [1, 2, 12].

The extent and accuracy of such knowledge and attitude are especially crucial for healthcare providers. In the case of vitamin D, manifestations of its deficiency may be expressed via loss of homeostasis in several systems of human physiology [1, 12]. It could be difficult for laypersons to acknowledge their deficiency and the link between vitamin D inadequacy and their symptoms. Consequently, the role of detection and recognition of vitamin D deficiency falls onto the physicians of both general and specialized practices. Moreover, study reports also suggested that along with general physicians, dentists also play a significant role to provide primary care [13, 14].

Overall, for any health-related issues, doctors are universally deemed as one of the most reliable sources of information and advice [15]. Therefore, the accuracy of the knowledge and attitude of the medical practitioners (general physicians and dentists) regarding the relationship between sunlight and vitamin D is extremely important to understand.

Despite its crucial importance, studies on the knowledge and attitude of medical practitioners regarding vitamin D deficiency and the relevant factors are scarce and also limited in scope. Yet all of these studies were carried out with a small group of medical students or practitioners (sample sizes ranged from 93 to 385), focusing solely on a specific institution [16–20]. Most importantly, none of the studies published in refereed journals focused explicitly on the participants' knowledge and attitude towards sunlight exposure concerning the production of vitamin D.

Since healthcare professionals are often regarded as the primary source of health-related knowledge for the general public, this study aims to evaluate their knowledge and attitude towards sunlight and their awareness regarding the clinical and epidemiological aspects of vitamin D deficiency.

## Methods

### Study setting

A cross-sectional descriptive study was conducted among the personnel directly involved in providing healthcare in Bangladesh – registered physicians (having full license of practice from Bangladesh Medical and Dental Council (BMDC), intern doctors, and senior medical students (4th and 5th-year students studying in medical and dental colleges). As part of the degree requirements, these senior students are routinely deployed in the hospital wards and they comprise a crucial component of the healthcare delivery system. Their roles are more important in settings where the hospitals need to cater to patients beyond their capacity with a fewer number of doctors; in places like Bangladesh.

### Sampling

There are approximately 115,000 doctors and dentists registered with the Bangladesh Medical and Dental Council (BM and DC), the regulatory body for doctors and medical education [21]. In addition, approximately 10,000 medical and dental students get enrolled per year to pursue their bachelor's degrees in 128 medical and dental colleges in Bangladesh [22, 23]. To obtain a confidence interval of 95% with a 5% precision level, the calculated minimum sample size was 369 for this study (the prevalence was considered as 40% based on previously published literature) [18, 20, 24]. For a collection of the desired number of responses, medical and dental colleges were selected from the databases of the Directorate General of Health Services (DGHS) following a stratified approach. Based on the local demographic diversity among the target population, attempts were made to keep proportional representations from medical vs. dental colleges, public vs. private colleges, and colleges from both urban vs. regional settings. Data collection for this study was carried out from October 2019 to February 2020.

### Instrument

The Strengthening the Reporting of Observational Studies in Epidemiology (STROBE) checklist was followed (Supplementary file 1) at various stages of this study. A structured questionnaire with close-ended questions was drafted to check the knowledge, attitude, and practices (KAP) of the healthcare providers. Some related KAP studies previously published in the literature were consulted before the local context and the study objective was considered to customize the questionnaire [16–20, 25, 26]. To increase the validity of our study, a series of focused-group discussions were then arranged with physicians, public health experts, medical students, and statisticians to finalize the questionnaire in line with the study objectives. The participants of these discussions were later excluded from the sampling. The final version of the questionnaire consisted of 12 questions; 6 were on knowledge, 5 were on attitude, and another was on their practice.

Knowledge-based questions were related to the diseases and symptoms associated with vitamin D deficiency; the best suitable time to get vitamin D from direct sunlight; the required minimum duration to get adequate vitamin D from sunlight; the average prevalence of vitamin D insufficiency in Bangladesh; and the groups of the population at high risk of vitamin D deficiency. Attitude-related questions, on the other hand, were related to the possible health effects of regular sunlight exposure; the potential level of UV radiation in the sunlight; the necessity to use sunscreen to avoid the adverse health effects of direct sunlight; and the potential intervention measures to address vitamin D deficiency. In addition, considering the medical practitioners could themselves be at risk, a question was included on whether they checked their own vitamin D levels ever.

Special attention was given to avoiding leading questions and double-barrel questions. The questionnaire was prepared both in Bengali and English. Backward and forward translations were done separately to ensure no change of meaning occurred. The sequence of the questions was shuffled to avoid any answering bias. No personally identifiable information (PII) was collected.

### Method of data collection

The self-reported data collection regime lasted from October 2019 to February 2020. Written permission was obtained from the target institutions before data collection. Schedules were fixed in advance for the individual institutions upon consultation with the respective authority when a large group of students or physicians were involved.

All the data-collection events occurred between Sunday to Thursday (except government holidays) in presence of either one of the co-investigators of this study or the pre-trained volunteers (senior medical students). In accordance with our scheduled date, two to three members of our research team attended the institutions. Following that, we visited the designated room (conference room or classroom) with the institution representative (faculty member or designated administrative staff), where our study participants were present. None of our participants were informed in advance about the objective of the study; only a 'health-related survey' was mentioned. The potential respondents were briefed on the answering procedures before the paper-based questionnaires were distributed among the participant. Precautions were taken to limit any verbal or non-verbal communications among the participants during the survey.

### Statistical analysis

Microsoft Excel 2019, was used for descriptive statistical analysis (percentage and frequency), and SPSS (version 25) was used for inferential statistical procedures to find out chi-square (χ2) and *p*-value. In addition, *p*-value < 0.05 considered as significant [27].

## Results

A total of 2,242 responses were collected from 15 medical institutions (12 medical colleges vs. 3 dental colleges; 11 urbans vs. 4 regionals; 9 public vs. 6 private institutions). Later, 52 responses were removed due to taking help from others during the survey, and for incomplete submissions. Finally, 2190 responses were eligible for further analysis. Among these, 400 were registered physicians, 457 from intern doctors, and 1333 were students (957 from medical colleges, and 376 from dental colleges).

### Participant’s attitude regarding sunlight exposure

**Fig. 1:**
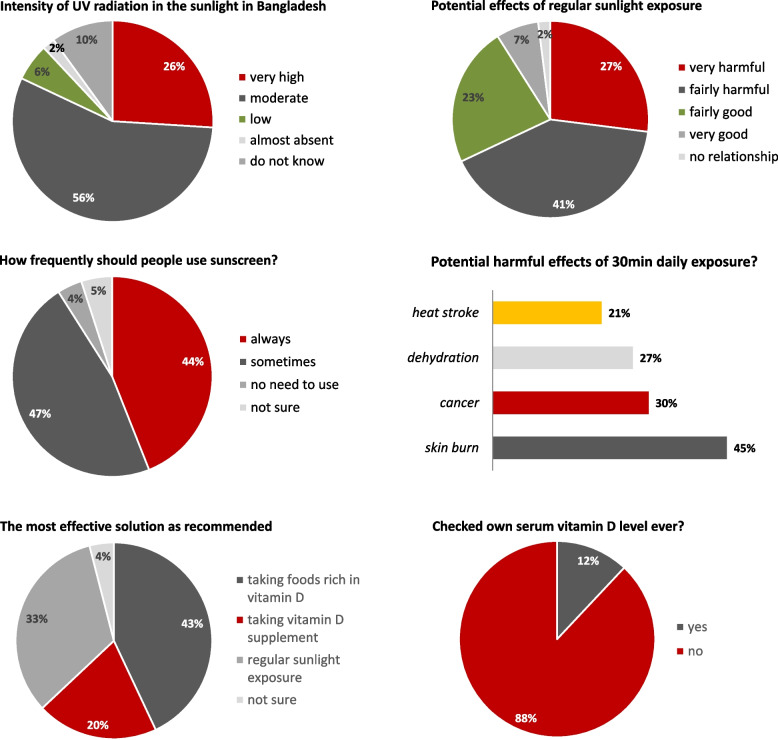
Overall attitude of the healthcare providers in Bangladesh was tested with the questions regarding possible extent of UV radiation in the sunlight in Bangladesh, potential health effects of regular exposure, recommendation regarding sunscreen usage, and if the respondents ever checked their own serum vitamin D ever. Multiple answers could be chosen for the question regarding potential harmful effects of 30-min daily exposure in the context of Bangladesh

When the participants were asked about the potential effects of regular sunlight exposure in the context of Bangladesh, 68% of all the respondents thought the effect would be harmful (27% thought this would be very harmful (Fig. 1), while 41% believed it would be fairly harmful. On the other side, 30% of the total respondents thought the effect of regular sunlight exposure would be good, while 2% of the respondents thought there was no relationship at all between sunlight exposure and human health (*p* < 0.001).

Among the list of possible harmful health effects of spending half an hour daily under direct sunlight for the Bangladeshi population (where multiple answers could be chosen), the highest number of respondents chose reduced skin brightness (58%), followed by skin burn (45%), risk of cancer (30%), dehydration (27%), and heat stroke (21%) (*p* < 0.001).

Only 12% of the respondents checked their vitamin D levels at least once (*p* < 0.001) (Fig. 1). When asked about the potential intervention measure to ensure adequate vitamin D in the Bangladeshi population, ‘taking foods rich in vitamin D’ was the most preferred among the respondents (43% chose this), followed by ‘regular sunlight exposure’ (by 33%), and ‘taking vitamin D supplements (by 20%) (*p* < 0.001).

Regarding the intensity of UV radiation in the sunlight in Bangladesh, 26% responded it was very high (Fig. 1), while the majority of the participants (52–57% across the three respondent groups) thought the level was 'moderate'; 10% reported that they did not know about the level of UV radiation in Bangladesh (*p < *0.001). Further insight into the comparison of attitudes among the different demographic groups can be found in Table 1.

**Table 1:** Participants’ attitude regarding sunlight exposure across different demographic strata has been compared. In response to the questions, here we presented the number of participants (n) alongside frequency percentage (%). A significant difference in attitude was observed, as suggested by *p*-value of less than 0.001

Questions asked to the respondents	Answer options (checkbox)	Registered doctors *n* = 400 (%)	Intern doctors *n* = 457 (%)	MBBS students (4^th^ / 5^th^ year); *n* = 957 (%)	BDS students (4^th^ / 5^th^ year); *n* = 376 (%)	χ2	*p-*value
*In general, what is the level of ultraviolet (UV) radiation in sunlight in Bangladesh?*	Very High	96 (24%)	165 (36%)	211 (22%)	105 (28%)	89.623630	< 0.001
	Moderate	228 (57%)	238 (52%)	526 (55%)	220 (61%)		
	Low	40 (10%)	18 (4%)	57 (6%)	19 (5%)		
	Almost absent	12 (3%)	9 (2%)	19 (2%)	4 (1%)		
	Do not Know	24 (6%)	27 (6%)	144 (15%)	23 (6%)		
*In your opinion, what can be the potential effects of regular sunlight exposure on the health of Bangladeshi people?*	Very harmful	88 (22%)	128 (28%)	258 (27%)	128 (34%)	36.343289	< 0.001
	Fairly harmful	188 (47%)	174 (38%)	373 (39%)	150 (40%)		
	Fairly good	76 (19%)	123 (27%)	230 (24%)	64 (17%)		
	Very good	36 (9%)	23 (5%)	77 (8%)	23 (6%)		
	There is no relationship	12 (3%)	9 (2%)	19 (2%)	11 (3%)		
If Bangladeshi people spend half an hour daily in direct sunlight, what could be the possible harmful effects?*	Skin burn	116 (29%)	288 (63%)	392 (41%)	188 (50%)	126.940562	< 0.001
	Reduce brightness of the skin	268 (67%)	247 (54%)	555 (58%)	203 (54%)		
	Increasing risk of cancer	64 (16%)	123 (27%)	335 (35%)	147 (39%)		
	Dehydration	64 (16%)	151 (33%)	249 (26%)	128 (34%)		
	Heat stroke	64 (16%)	91 (20%)	182 (19%)	120 (32%)		
*In your opinion, what is the level of necessity for Bangladeshi people to use sunscreen to avoid the negative effects of direct sunlight?*	Should be used always	104 (26%)	224 (49%)	412 (43%)	229 (61%)	126.498326	< 0.001
	Can be used sometimes	252 (63%)	215 (47%)	431 (45%)	120 (32%)		
	No need to use at all	24 (6%)	5 (1%)	57 (6%)	8 (2%)		
	Not sure	20 (5%)	14 (3%)	57 (6%)	19 (5%)		
*Have you ever checked your own vitamin D level?*	Yes	48 (12%)	37 (8%)	115 (12%)	53 (14%)	26.264611	< 0.001
	No	332 (83%)	370 (81%)	756 (79%)	308 (82%)		
	Not sure	20 (5%)	50 (11%)	86 (9%)	15 (4%)		
*Considering the overall condition, which of the following options might be most effective to ensure adequate vitamin D level for Bangladeshi population?*	Taking fortified food containing vitamin D	156 (39%)	142 (31%)	431 (45%)	199 (53%)	71.664610	< 0.001
	Taking vitamin D supplement	76 (19%)	119 (26%)	172 (18%)	75 (20%)		
	Regular sunlight exposure	160 (40%)	178 (39%)	316 (33%)	79 (21%)		
	Not sure	8 (2%)	18 (4%)	38 (4%)	23 (6%)		

^*^Multiple answers could be chosen by the respondents

The attitude towards sunlight was grossly negative as demonstrated by 44% of the total respondents reporting that sunscreen should always be used in Bangladesh to avoid the negative effect of direct sunlight (Fig. 1); this negative attitude towards sunlight was significantly more prevalent (*p* < 0.001) among the younger respondents; (49% and 48% among the intern doctors and the students respectively compared to 26% of the senior registered doctors). However, variation of attitude among these different demographic groups was not significant in most cases, suggesting homogeneity of attitude among the Bangladeshi healthcare providers.

### Participants' knowledge about vitamin D deficiency

**Fig. 2:**
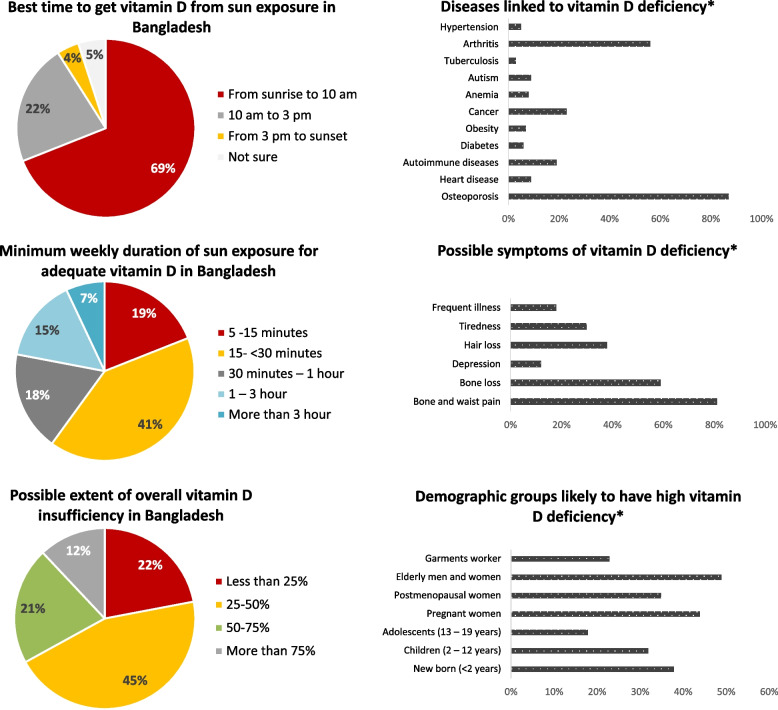
Overall knowledge of the healthcare providers regarding sun exposure and vitamin D was tested with the questions regarding the best suitable time to get vitamin D from sunlight exposure in Bangladesh, minimum weekly duration of sun exposure for adequate vitamin D, diseases linked to vitamin D deficiency, possible symptoms of vitamin D deficiency, possible extent of overall vitamin D insufficiency in Bangladesh, and demographic groups likely to have high vitamin D deficiency. The bar-charts (*) represent data collected with questions where multiple answers could be chosen

Among all the respondents, 70% reported that the best time for the production of vitamin D from sunlight exposure in Bangladesh would be from sunrise to 10 am, while 22% believed ‘10 am to 3 pm, and 4% thought it was 'after 3 pm’ as the right time; 5% were 'not sure' about the correct timing (*p* < 0.001) (Fig. 2) Regarding how much weekly sunlight exposure can be considered sufficient (for the production of serum vitamin D) for the Bangladeshi population, 60% of the participants chose ‘less than 30 min, 41% thought 'between 15 to 30 min and 19% believed only '5 to 15 min as appropriate. On the contrary, 18% thought ‘30 min to one hour would be appropriate, while 15% believed that 'between 1 and 3 h would be necessary (*p* < 0.001).

Among the symptoms of vitamin D deficiency, 'bone and waist pain' was the most commonly identified symptom (by 81% of the total participants) associated with vitamin D deficiency. However, lesser percentages of the respondents could identify the other symptoms associated with such deficiency – bone loss (59%), hair loss (38%), tiredness (30%), frequent illness (18%), and depression (12%) (*p* < 0.001).

Regarding the health effects of vitamin D deficiency, the most common diseases identified by the study participants were osteoporosis (87%), arthritis (56%), cancers in general (23%), and autoimmune diseases (19%). On the contrary, a lot less percentage of respondents could identify the link of such deficiency with heart disease (9%), autism (9%), anemia (8%), diabetes (6%), obesity (6%), hypertension (5%), and tuberculosis (3%). Interestingly 4% of the respondents reported they were 'not sure' about any of these diseases (*p* < 0.001).

Among the demographic groups at risk of vitamin D deficiency, the elderly men and women, children and newborn babies (< 2 years), and pregnant women were identified by 49%, 39%, and 44% of respondents (*p* < 0.001). In the case of vitamin D insufficiency, two-thirds (67%) of the respondents had a view that the prevalence of vitamin D insufficiency among the adult population in Bangladesh would be < 50% as opposed to only 33% of the respondents identifying that more than half of the population in Bangladesh could be affected (*p* < 0.001).

Similar to what was observed for attitude towards sunlight, the knowledge related to timing and duration of sun exposure to safely get adequate vitamin D, the symptoms and potential effects of vitamin D deficiency, and the extent of prevailing vitamin D deficiency across different age-groups was mostly comparable across the different demographic groups of healthcare providers in Bangladesh (Table 2).

**Table 2:** Knowledge of the participants regarding sun exposure and vitamin D across different demographic strata has been compared. Here, we provide the total number of participants (n) together with the frequency percentages (%) in response to the questions. A significant difference in knowledge was observed, as indicated by a *p*-value of less than 0.001

Questions asked to the respondents	Answer options (checkbox)	Registered doctors *n* = 400 ( %)	Intern doctors *n* = 457 (%)	MBBS students (4th / 5th year); *n* = 957 (%)	BDS students (4th / 5th year); *n* = 376 (%)	χ2	*p-*value
*Which is the best time to get vitamin D from direct sunlight in Bangladesh?*	From sunrise to 10 am	296 (74%)	320 (70%)	603 (63%)	293 (78%)	58.393096	< 0.001
	10 am to 3 pm	72 (18%)	101 (22%)	239 (25%)	64 (17%)		
	From 3 pm to sunset	8 (2%)	9 (2%)	67 (7%)	8 (2%)		
	Not sure	24 (6%)	27 (6%)	48 (5%)	11 (3%)		
*What should be the required minimum duration of direct sunlight exposure per week for Bangladeshi population to get adequate vitamin D?*	5 -15 min	68 (17%)	101 (22%)	163 (17%)	83 (22%)	93.559972	< 0.001
	15- < 30 min	208 (52%)	183 (40%)	354 (37%)	135 (36%)		
	30 min – 1 h	56 (14%)	55 (12%)	220 (23%)	68 (18%)		
	1 – 3 h	44 (11%)	87 (19%)	172 (18%)	41 (11%)		
	More than 3 h	24 (6%)	32 (7%)	48 (5%)	49 (13%)		
Which of the following are the symptoms associated with vitamin D deficiency?*	Bone and waist pain	328 (82%)	379 (83%)	756 (79%)	312 (83%)	71.025201	< 0.001
	Bone loss	224 (56%)	265 (58%)	526 (55%)	274 (73%)		
	Depression	56 (14%)	64 (14%)	96 (10%)	49 (13%)		
	Hair loss	140 (35%)	201 (44%)	373 (39%)	120 (32%)		
	Tiredness	108 (27%)	142 (31%)	316 (33%)	98 (26%)		
	Frequent illness	120 (30%)	69 (15%)	163 (17%)	53 (14%)		
For which of the following diseases, deficiency of vitamin D can be a risk factor?*	Osteoporosis	356 (89%)	407 (89%)	813 (85%)	338 (90%)	187.214245	< 0.001
	Heart disease	44 (11%)	27 (6%)	86 (9%)	38 (10%)		
	Autoimmune disease	56 (14%)	105 (23%)	163 (17%)	83 (22%)		
	Diabetes	24 (6%)	14 (3%)	77 (8%)	19 (5%)		
	Obesity	28 (7%)	32 (7%)	67 (7%)	19 (5%)		
	Cancer	20 (5%)	174 (38%)	230 (24%)	83 (22%)		
	Anemia	48 (12%)	41 (9%)	67 (7%)	15 (4%)		
	Autism	28 (7%)	41 (9%)	105 (11%)	26 (7%)		
	Tuberculosis	4 (1%)	5 (1%)	57 (6%)	8 (2%)		
	Arthritis	212 (53%)	320 (70%)	526 (55%)	184 (49%)		
	Hypertension	16 (4%)	9 (2%)	67 (7%)	19 (5%)		
	Not sure	16 (4%)	14 (3%)	38 (4%)	15 (4%)		
Which group of Bangladeshi population is likely to suffer from vitamin D deficiency?*	New born (< 2 years)	100 (25%)	219 (48%)	354 (37%)	165 (44%)	199.123366	< 0.001
	Children (2 – 12 years)	84 (21%)	101 (22%)	354 (37%)	165 (44%)		
	Adolescents (13 – 19 years)	48 (12%)	91 (20%)	163 (17%)	86 (23%)		
	Pregnant women	216 (54%)	201 (44%)	373 (39%)	169 (45%)		
	Postmenopausal women	116 (29%)	137 (30%)	392 (41%)	117 (31%)		
	Elderly men and women	216 (54%)	251 (55%)	488 (51%)	120 (32%)		
	Corporate employee	124 (31%)	91 (20%)	201 (21%)	56 (15%)		
	Garments worker	96 (24%)	110 (24%)	230 (24%)	71 (19%)		
	Heart patients	16 (4%)	14 (3%)	38 (4%)	26 (7%)		
*What could be the average prevalence of vitamin D insufficiency in Bangladesh?*	Less than 25%	88 (22%)	73 (16%)	230 (24%)	94 (25%)	32.893841	< 0.001
	25–50%	204 (51%)	215 (47%)	431 (45%)	147 (39%)		
	50–75%	68 (17%)	101 (22%)	182 (19%)	98 (26%)		
	More than 75%	40 (10%)	69 (15%)	115 (12%)	38 (10%)		

^*^Multiple answers could be chosen by the respondents

## Discussion

Responses recorded in this study provide a comprehensive insight into the knowledge and attitude about the metabolism of vitamin D and its physiological role (which is usually formed during the early years of medical school [28]) among the healthcare professionals in Bangladesh. To the best of our knowledge, this study is among one of the most comprehensive investigations encompassing a diverse population of healthcare providers an also due to its high sample size (*n* = 2,190). Results of this study highlight that an overall negative attitude towards sunlight exposure could be predominant among some of the medical practitioners in Bangladesh (evident from 68% of respondents believing that regular sunlight exposure in Bangladesh would be harmful contrary to 30% deeming this as good; Fig. 1). This was further evidenced by a high percentage of the respondents identifying skin burn and cancer as potential outcomes of 30-min daily exposure (45% and 30% respectively; Fig. 1). But, is such a negative attitude among the medical community in Bangladesh supported by strong evidence?

Bangladesh has a subtropical climate where, unlike the tropical countries, sunlight intensity remains relatively mild. The monthly average of the highest daily UV index ranges from 6 to 7 throughout the year which is not as high as it is observed in many other tropical countries [29]. Moreover, this range (6 to 7) reflects the daily highest levels that last for a short duration of a day. Furthermore, the UV index is a physical parameter. Any quantitative measurement of its effects on various aspects of human health depends on other factors (e.g., skin complexion, clothing practices, etc. [30]). The effect any UV exposure could cause to fair skin in 15 min could take up to hours for people with darker skin because of melanin (which is responsible for skin darkness) absorbing the UV radiation [3, 31]. In this regard, the South Asian population has generally dark skin complexion belonging to skin-type IV and V according to the Fitzpatrick complexion scale [32, 33]. This most likely explains Bangladesh ranking 183rd globally on the list for the prevalence of melanoma [34], and also sunburn is extremely rarely reported in this region. These facts indeed bolster the argument against the hypothesis that the intensity of UV radiation in Bangladesh will have highly negative effects on human health. Therefore, the perception of the level of UV radiation in Bangladesh being 'very harmful' and extrapolating this to conclude that direct sunlight exposure would bring harmful to very harmful effects to human health could most likely be an overstatement. In this regard, further research on the dose–response relationship and quantitative risk assessment in the context of Bangladesh and South Asia could be particularly helpful to gain further insight. But for now, could such a negative attitude among the healthcare providers have larger and more direct implications for the wider community?

Only 22% of the participants of this study identified the time ‘from 10 am to 3 pm as the best time for the production of vitamin D in Bangladesh (Fig. 2), whereas 69% of the respondents believed ‘before 10’ am as the best time. While there is no published literature regarding this timing in the context of Bangladesh (or any neighboring country), the solar intensity in the morning and the afternoon (after 3 pm) remains low to mild due to its mostly subtropical climate [35]. This means UV-B intensity remains very low during this time, which starts getting more intense as it approaches mid-day. Along with this, taking the generally dark complexion of the Bangladeshi population (skin types IV and V) [32, 33], net absorption of UV-B (even after direct exposure) by an average Bangladeshi in the morning would likely be really low. However, this scenario would change around mid-day time (10 am to 3 pm) when the level of UV-B in the sunlight would be maximum. Hence, it is highly likely that this time of the day comprises the best time for the production of vitamin D from direct sunlight exposure, which is also strongly supported by the literature [1, 3, 12]. As such, the hypothesis that the time between sunrise and 10 am is the best time to get vitamin D from sunlight, particularly in the context of Bangladesh, is most likely not correct. While the lack of research data regarding the best time to get vitamin D from sun exposure (preferably across different seasons throughout the year) highlights a significant knowledge gap, the majority of the medical practitioners identifying the wrong time also needs to be addressed.

On a similar note, regarding the minimum weekly duration to get enough vitamin D from sunlight, a clear majority of the respondents (60%) thought < 30 min of direct sunlight would be adequate for the Bangladeshi population. While any specific guideline or clear consensus among the researchers is missing in this regard, Holick et al. (2007) suggested 5 to 30 min of sunlight exposure twice a week could often be sufficient [12]. However, this recommendation was based on studies conducted on people with lighter skin completion, and as such, it has been argued later that this could be a significant underestimation for many other populations (as the required weekly exposure could be significantly higher when other aspects like darker skin complexion and clothing practices are considered [36]). While further research is warranted to determine the minimum duration more precisely, considering the darker skin complexion of the South Asians in general and heavier clothing practices in this region for cultural and religious reasons (like in Bangladesh [37]), it would be safer to believe that the minimum required weekly exposure would almost certainly exceed 30 min. As such, the majority of the healthcare providers thinking (and perhaps communicating with the mass people) that < 30 min of weekly sunlight exposure would be adequate is highly likely to be inaccurate. More specific experimental studies for a clearer understanding of the dynamics of serum vitamin D production as a result of sunlight exposure under varying conditions would be highly beneficial in this regard.

However, we hypothesize that the healthcare professionals undermining the necessity for longer duration constitutes a significant source of misinformation for the wider community, which might be one of the contributors to the high prevalence of vitamin D deficiency in Bangladesh and this could be true for South Asia overall [5–7]. The absence of such studies (regarding sunlight and vitamin D) in the other South Asian regions, therefore, represents a key research gap regarding the accuracy of knowledge and attitude.

Among the symptoms of vitamin D deficiency, those having obvious relation to Calcium (Ca) metabolism (like 'bone and waist pain' and bone loss) were more readily identified as the symptoms of vitamin D deficiency, while those having a less obvious connection to Ca-metabolism (like hair loss, tiredness, frequent illness, and depression) were identified by only a small percentage (Fig. 1). A similar trend was observed for the potential long-term effects of vitamin D deficiency; osteoporosis and arthritis were identified by the majority of the respondents, the other potentially associated diseases like cancer, diabetes, heart disease, hypertension, anemia, obesity, autism, etc. were identified by very few (< 10%) respondents in each case. This leads to a hypothesis that the medical practitioners in Bangladesh might be more focused on Ca-metabolism while the other critical roles of vitamin D might be significantly overlooked. Indeed, this has been reported in studies conducted in other countries as well [18, 38]. So, what might be behind the suboptimal level of knowledge among healthcare professionals in general?

The majority of the respondents (67%) being unaware of the very high prevalence of vitamin D deficiency in Bangladesh as well as in the whole South Asian region across all age groups could be related to this [5–7]. Despite such high prevalence, a general lack of awareness about the seriousness of this problem perhaps explains why only 12% of the healthcare providers have checked their serum vitamin D level ever (Fig. 1).

Finally, taking food that is rich in vitamin D was preferred over regular sunlight exposure as a better intervention to mitigate widespread vitamin D deficiency in Bangladesh (Fig. 1). In this regard, when the poor socioeconomic demography of Bangladesh is considered, taking vitamin D-rich foods in sufficient quantity (yet on regular basis) by the mass people in Bangladesh might be practically challenging for a large portion of the community. Also, focusing mostly on dietary sources for vitamin D can undermine the more sustainable option, since most of the serum vitamin D (as high as up to 90%) is known to be produced in the skin from regular sun exposure only [39], which is abundant in Bangladesh throughout the year. Furthermore, the vitamin D precursor (25-hydroxy vitamin D) produced from sunlight has a higher half-life compared to when it is absorbed from dietary sources or vitamin D supplements [40, 41]. Therefore, from a more practical perspective, we argue that regular sunlight exposure can be a good, and perhaps sustainable, option at a mass scale for the wider community in Bangladesh and other countries with similar geographic and socioeconomic contexts.

The major focus of our study was how the medical community views the health implications of regular sunlight exposure, particularly in the context of getting vitamin D for individuals and the wider community. Regarding the knowledge related to the metabolism and pathological aspects of vitamin D, our overall findings conform to a handful of other published studies conducted in China, Saudi Arabia, and Pakistan. In Saudi Arabia, a study conducted among general physicians and their findings point out that knowledge and practice need to be improved regarding vitamin D [17]. In China, a study was conducted among medical students and the result showed that there is little knowledge and unfavorable behaviors regarding vitamin D among their study participants [19]. In Pakistan, a study was conducted among medical students and their findings also highlighted the need for improved knowledge about vitamin D and its metabolism among study participants [16]. As such, our study was unique due to it addressing the knowledge and attitude regarding the potential health implication of sun exposure, a much-required practice, particularly in countries with a high prevalence of vitamin D deficiency. Furthermore, this study encompassed a wider spectrum of healthcare providers (from junior to senior level) yet was conducted on a large number of participants.

### Limitations

Due to the major focus of this study being the knowledge and attitude regarding sunlight exposure to getting vitamin D, many of our questions were unique. Therefore, we could not fully compare our participants' answers (regarding sun exposure) to other studies. Also, we could not perform any demographic analysis as personal information (age, gender, etc.) was not collected due to privacy concerns. In addition, caution might be practiced while interpreting the knowledge level, particularly in case of the vitamin D deficiency related diseases as the causal associations are still being debated. Finally, despite our best efforts to keep the samples representative (by considering the stratifications that exist), we acknowledge that true randomization could not be achieved. Indeed, a very high number of responses (higher than any other published studies according to the best of the knowledge of the authors) were collected to offset any potential sampling bias. However, caution should still be maintained before generalizing the conclusions for the entire medical community in Bangladesh and beyond.

## Conclusion

To our knowledge, this is the first study on knowledge, attitude, and practices among the full spectrum of the medical community (senior students, intern doctors, and registered physicians) regarding the relationship between sunlight exposure and vitamin D. This study offers a strong reminder that continuous efforts should be taken to assess and improve the accuracy of knowledge and attitude of the medical practitioners regarding the issues related to public health. Highlights of our findings reveal the over-estimated fear of health risks and widespread negative attitude towards sunlight exposure. This study has also identified that such negative attitudes were most likely based on incomplete (and often inaccurate) knowledge regarding sunlight exposure. Additionally, this study has highlighted the urgent need to address key knowledge gaps regarding the timing and duration of safe sun exposure practices. Overall, this study has underscored the need to prioritize initiatives to ensure evidence-based knowledge among medical practitioners regarding the symptoms and effects of vitamin D deficiency as well as about the very high prevalence of this deficiency among the wider community. The insights gained from this study can be generalizable to many other countries having comparable socio-demographic settings. Being novel in multiple aspects, we believe, this study would make a significant impact to ensure better policy-making globally by facilitating better health communication.

## Supplementary Information

**Additional file 1. **STROBE Statement—Checklist of items that should be included in reports of cross-sectional studies.

## Data Availability

Only aggregated summaries of the data are provided in this manuscript. However, all data generated in this study can be made publicly available on request. Please contact the corresponding author for any kind of data request.
